# A Systematic Review of Tobacco Smoking Prevalence and Description of Tobacco Control Strategies in Sub-Saharan African Countries; 2007 to 2014

**DOI:** 10.1371/journal.pone.0132401

**Published:** 2015-07-10

**Authors:** Rachel Brathwaite, Juliet Addo, Liam Smeeth, Karen Lock

**Affiliations:** 1 Department of Non-communicable Disease Epidemiology, Faculty of Epidemiology and Population Health, London School of Hygiene and Tropical Medicine, London, United Kingdom; 2 Department of Health Services Research and Policy, Faculty of Public Health and Policy, London School of Hygiene and Tropical Medicine, London, United Kingdom; University of Tolima, COLOMBIA

## Abstract

**Objective:**

To systematically review current smoking prevalence among adults in sub-Saharan Africa from 2007 to May 2014 and to describe the context of tobacco control strategies in these countries.

**Data Sources:**

Five databases, Medline, Embase, Africa-wide Information, Cinahl Plus, and Global Health were searched using a systematic search strategy. There were no language restrictions.

**Study Selection:**

26 included studies measured current smoking prevalence in nationally representative adult populations in sub-Saharan African countries.

**Data Extraction:**

Study details were independently extracted using a standard datasheet. Data on tobacco control policies, taxation and trends in prices were obtained from the Implementation Database of the WHO FCTC website.

**Results:**

Studies represented 13 countries. Current smoking prevalence varied widely ranging from 1.8% in Zambia to 25.8% in Sierra Leone. The prevalence of smoking was consistently lower in women compared to men with the widest gender difference observed in Malawi (men 25.9%, women 2.9%). Rwanda had the highest prevalence of women smokers (12.6%) and Ghana had the lowest (0.2%). Rural, urban patterns were inconsistent. Most countries have implemented demand-reduction measures including bans on advertising, and taxation rates but to different extents.

**Conclusion:**

Smoking prevalence varied widely across sub-Saharan Africa, even between similar country regions, but was always higher in men. High smoking rates were observed among countries in the eastern and southern regions of Africa, mainly among men in Ethiopia, Malawi, Rwanda, and Zambia and women in Rwanda and rural Zambia. Effective action to reduce smoking across sub-Saharan Africa, particularly targeting population groups at increased risk remains a pressing public health priority.

## Background

The prevalence of smoking differs widely between populations across the world.[1, 2] Many factors are known to influence smoking prevalence and trends in prevalence, from individual level factors such as education level, to country-level factors such as national economic development and implementation of tobacco control policies.[3, 4] The highest prevalence of tobacco consumption has previously been found in high-income Western European countries, with a 37% prevalence among men and 25% among women.[1, 5] During the years 1990 to 2009, cigarette consumption in Western Europe declined by approximately 26%. Simultaneously during the same period, cigarette consumption in Africa and some middle Eastern countries increased by 57%.[1] Sub-Saharan Africa is considered to be in stage one of the tobacco epidemic continuum.[6] One characteristic of this first stage is a predominantly higher prevalence of smoking among men than among women. The estimated prevalence of smoking in sub-Saharan in 2010 was 14% in males and 2% in females, which supported this first stage criteria.[5] A previous systematic review published in 2006 on the prevalence of smoking among adult populations in sub-Saharan Africa was conducted using studies published before 2005.[7] In that review, men smoked more than women in all the sub-Saharan African countries represented. However, most of the studies in the review used study samples that were not representative of the national general populations, since only 6 national studies were amongst those included of which 4 were from South Africa and one each from Malawi and Zambia. The aim of this study was to systematically review the literature in order to provide contemporary estimates of the prevalence of smoking among adults in sub-Saharan Africa from January 2007 to May 2014. We also described country-level tobacco policies across sub-Saharan Africa to give some context and background about what is occurring to tackle smoking prevalence.

## Materials and Methods

### Search strategy for relevant articles

In May 2014, five databases of Medline, Embase, Africa-wide Information, Cinahl Plus and Global Health were searched using a strategy merging terms for sub-Saharan Africa and the countries in them, smoking, and terms for types of studies. The review protocol forms part of the PhD thesis of one of the authors (RB) but has not been separately published. The S1 Appendix provides details of the search strategy used. All of the references cited by the included studies were screened for other relevant articles.

### Inclusion criteria

The titles and abstracts of the results generated from the searches were screened using the following inclusion criteria:

1. The participant recruitment strategy of each study must have involved sampling from a representative sample of the general population, and/or from a rural or urban area in a country in sub-Saharan Africa
2. The sample size must comprise more than 1000 participants
3. The research must have estimated the prevalence of current smoking in the sample
4. Data must have been collected during 2007 and May 2014

The searches were limited by date of publication from 2007 to May 2014, but not by language of publication. Research studies conducted in a sub-Saharan African population in another part of the world, or using unrepresentative population samples within Africa (e.g. only adolescents or older populations, employed workers, hospital patients or community-based unrepresentative samples) were excluded. (Refer to S2 Appendix which indicates the reasons why studies were excluded from the review).

Potentially relevant articles were obtained after screening titles and abstracts, and decisions on including studies were made after reading the full texts. Data were extracted following a standard protocol and using standard data collection forms and checklist by a single reviewer (RB) with uncertainties resolved by discussion with the co-authors.

### Appraisal of study validity

To assess the quality of individual studies and to determine whether it was included, the following criteria were used.

1. Likely representativeness of the general population: whether nationwide sampling frames or more locally based populations were used; whether random population sampling, multi-stage sampling, or all participants were recruited from the entire sampling area.
2. In addition to a good representative sample, we looked at whether the study had either a large sample size and or a high response rate. If the response rate was not reported in the study, we assessed whether both the sample size and the recruitment method was sufficient to indicate the study was of good quality.

### Rationale for inclusion criteria

We chose to apply the above mentioned inclusion criteria to minimize the presence of selection bias affecting the results obtained.[8] We ensured that the included studies addressed the question of interest and provided evidence on the prevalence of smoking in a sub-Saharan African area. We believed that the way in which participants were chosen to participate, and the participation rates can be a major source of selection bias and therefore these methods were assessed before a study was included. As a result only studies that ensured that the recruited individuals were representative of the target population and or the selection process involved a form of randomization were included. In combination with having a fairly random recruitment method, which should yield a representative sample, we decided that studies with a minimum sample size of 1000 would have a more precise estimate of current smoking in the target population due to less sampling error compared to a smaller sample size.

### Search for preventative measures

In an attempt to explore some contextual factors which might be influencing the prevalence of current adult smoking among countries in sub-Saharan Africa, we set out to describe the preventative measures that were in place at the country level to reduce tobacco consumption. We examined documentary evidence of the implementation status of Articles 6 and 13, the last two components of the MPOWER policy package, which falls under the Demand Reduction measures of the World Health Organization Framework Convention on Tobacco Control (WHO FCTC) Treaty. [9, 10] These include whether the country has enforced bans on tobacco advertising, promotion and sponsorship and whether taxes on tobacco has been raised. We also examined some components of Article 16 under the Supply reduction measures of the Treaty provisions, which requires bans to be placed on sales to and by minors. The information on preventative measures was obtained from the Implementation Database of the WHO FCTC website.[2, 10] For each included country, we extracted data, where available, on the date of adoption of the WHO FCTC and recorded the status of the following: whether tax policies to reduce tobacco consumption were present; what proportion of the retail price of cigarettes comprised taxes; and whether there has been an increasing or decreasing trend in tobacco prices. Also under the tobacco advertising and sponsorship article, we gathered data on whether smoking was banned in public places and if comprehensive bans on all tobacco advertising, promotion and sponsorship were present. Under the supply reduction measures, prohibition on the sale of tobacco products from vending machines and of individually packaged or packets of small cigarettes were examined.[10]

### Responsibility of co-authors

All 4 authors were involved in the study design, and the writing of the paper. RB had the responsibility of screening the abstracts and full text articles, and selecting the articles for inclusion. LS, JA and KL provided advice on the review methods and were involved in any discussions to resolve uncertainties over study inclusion.

## Results

### Description of study populations and methods of measuring current smoking

**Fig 1:**
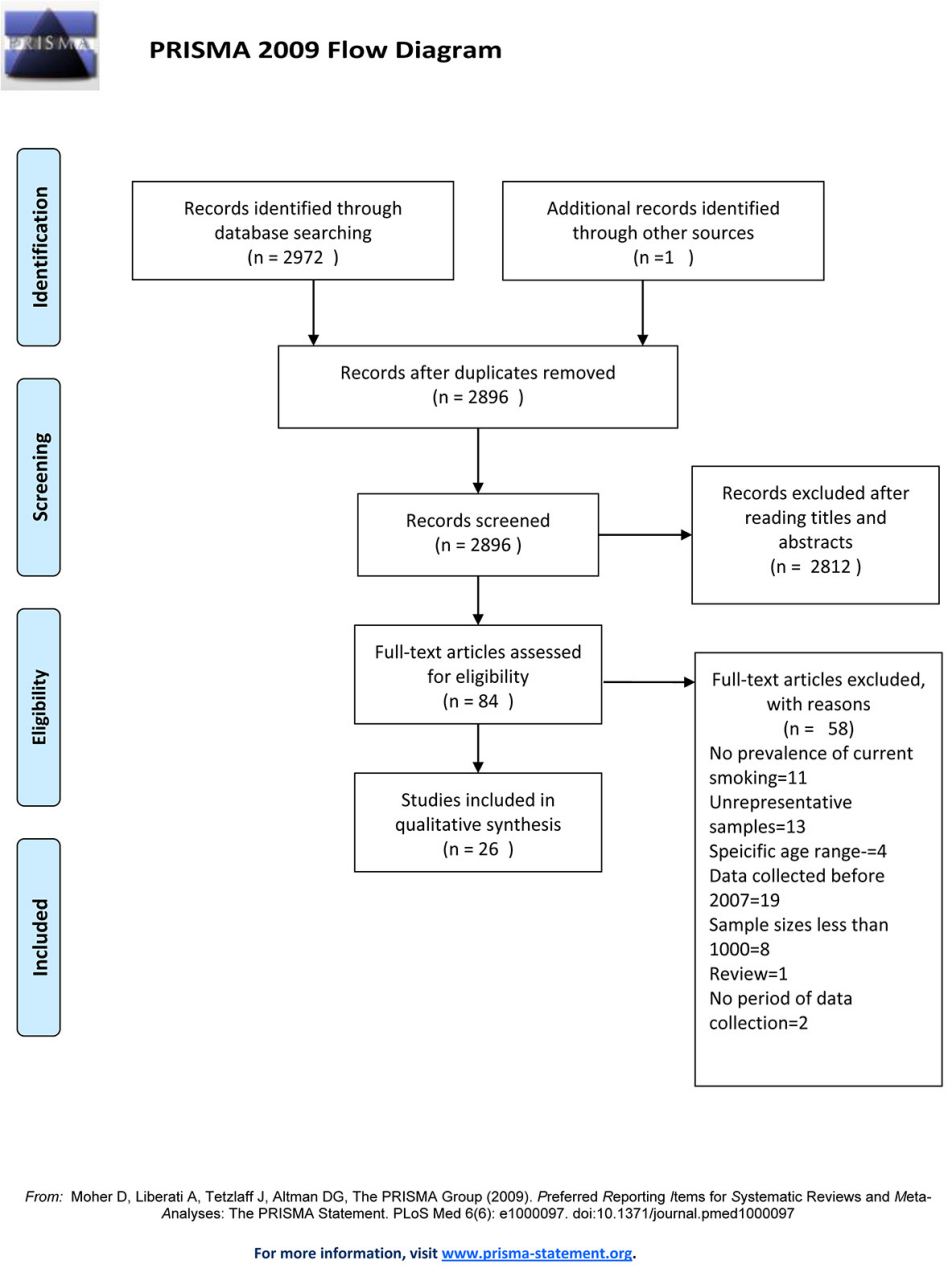
Flow Diagram of inclusion and exclusion of articles in systematic review.

26 papers were included in the review (Fig 1). These described 21 studies which represented research conducted in 13 countries of sub-Saharan Africa. The characteristics of study populations are presented in Table 1. Among these 13 countries, five were geographically located in the west (Ghana, Nigeria, Senegal, Sierra Leone, and Togo), one in the central region (Congo) and seven located in the eastern and southernmost regions of Africa (Ethiopia, Kenya, Malawi, Rwanda, Uganda, Zambia and South Africa). Estimates of the prevalence of current smoking obtained from the included studies represented the period 2007 to 2012, with sample sizes that ranged from 1,412 in Congo to 72,292 participants in Kenya.

**Table 1:** Characteristics of study populations of included studies.

Study ID	Country, year of data collection, reference	Age range	Median (IQR) Age	Mean (SD)	Total sample size	Response Rates %	Definition of current smoking as measured in the study
1	Congo, 2010,[29]	5–83	-	29 (16)	1412	93.4	Active smoking
2	Ethiopia, 2008–09,[26]	15–64	-	-	5500	81.3	Current smokerNo/Yes
3	Ghana, 2008,[11]	15–49	-	-	9484	98.9	Currently consumed cigarettes
4	Ghana, 2007–08,[12]	14–105	31	-	6258	88.0	Smokes at least 100 cigarettes in his or her lifetime and smokes nowadays
5	Kenya, March to July 2010,[30]	>18	35 (26,50)	-	4037	93.0	Tobacco smoking- Currently daily (within the last 30 days)
							Tobacco smoking- Currently but not daily (within the last 30 days)
6	Kenya, 2010,[19]	>18	-	33.4 (11.6)	2061	-	Current cigarette smokers
7	Kenya, 2011,[13]	>18	-	-	72292	-	Currently smoked at least 100 cigarettes and smoking at the time of the interview
							Smoked daily
8	Malawi, 2009,[15]	25–64	-	-	5206	94.5	Tobacco smokers
9	Nigeria, 2008,[16]	15–59	-	-	48871	98.3	Use of smoking tobacco- cigarette, pipe or other
10	Nigeria, 2007,[25]	25–64	-	43.8 (13.7)	1458	-	Prevalence of tobacco smoking
11	Rwanda, 2008–2009,[27]	15–80	-	38.3	1824	97.7	Currently smoking at least one cigarette per day
	(Huye- rural)	-	-	-	788	-	
	(Kigali- urban)	-	-	-	1036	-	
12	Senegal, 2010,[23]	>15	-	43.4 (17.8)	1424	-	Tobacco smokers- active smoking
13	Sierra Leone, 2009,[14]	25–64	-	-	4997	91.0	Current tobacco use (smoke and smokeless)
14	South Africa, 2008,[17]	>15	-	40 (16.7)	16800	69.0	Non-smoker or smoker
15	Togo, October 2009 to January 2010,[20]	>18	-	45 (10)	2000	-	Consumed at least one cigarette per day
16	Uganda, December 2008 to November 2009,[31]	>13	-	-	6663	65.6	Current cigarette smoker of both manufactured and local cigarettes
17	Uganda, 2011,[44]	>13	-	34.4	6867	93.9	Current daily smoking
18	Uganda, July to September 2012,[22]	>18	-	36.5 (15.2)	4142	-	Currently consume tobacco
19	Zambia, December 2008 to 2009,[21]	>25	-	-	2093 (total)	-	Current smoking- do you currently smoke any tobacco products such as cigarettes, cigars or pipes
	(Kasama- rural)	-	-	-	1198	-	
	(Kaoma- rural)	-	-	-	895	-	
20	Zambia, 2011,[28]	>25	-	-	1627	-	Current smoking- do you currently smoke any tobacco products such as cigarettes, cigars, or pipes
21	Zambia, 2008,[24]	>25	-	33.4 (11.6)	1928	-	Currently smoked cigarettes

SD- Standard deviation; IQR-Interquartile range;-Data not available

Among the studies, current smoking among adults was determined using five broad criteria. Some researchers measured either 1) smoking of tobacco including all its products or 2) cigarettes only, while some assessed 3) the frequency of current smoking, that is, either daily or occasionally or 4) regularly during a specific time period prior to the study. 5) A few research studies measured the quantity of consumption within this time period.

From the 26 studies, age-specific smoking prevalences estimates were only presented in a total of four studies that were conducted in Ghana (2),[11, 12] Kenya[13] and Sierra Leone.[14] From this limited data we observed that the prevalence of smoking increased as the age category increased then decreased slightly in the older age groups.

### Quality of evidence

Research using a nationwide sampling frame were conducted in Ghana,[11] Malawi,[15] Nigeria,[16] Sierra Leone,[14] South Africa,[17] and Uganda.[18] These studies therefore provided the strongest evidence on the prevalence of current smoking since the sampling methodology was the most representative of the general population area of the country. These studies had large sample sizes (4,997 in Sierra Leone,[14] 5,206 in Malawi,[15] 6,678 in Uganda,[18] 9,484 in Ghana,[11] 11,638 in South Africa[17] and 34,070 in Nigeria).[16] The response rates were also high among these national studies, with the lowest response rates observed in the Ugandan (65.9%)[18] and South African (69%) studies.[17] (Refer to S3 Appendix for supplementary data on details of the sampling methodology used in each study). In contrast high response rates were obtained in the studies in Sierra Leone (91%),[14] Malawi (94.5%),[15] Nigeria (98.3%)[16] and Ghana (98.9%).[11]

Other studies sampled from sub-regions of the population so the prevalence was less representative of the entire national population. However, the sampling methodology used ensured that the samples were representative of the area and that area was similar in characteristics to the general population. All studies used either multi-stage sampling strategies to ensure random selection of participants or census type surveys where all residents in areas were eligible to participate. In addition all studies recruited participants from a wide age range, apart from a few exceptions, most were from over 15 and 25 years up to age 64 which meant that most of the results were generalizable to not just a specific age group. Adequate sample sizes and good response rates were also observed among these sub-national samples.

Although response rates were not known for 9 studies, the methodology employed was of a high standard and the final sample size was large enough to justify the study of good quality to be included.[13, 19–25]

### Main findings

The prevalence of smoking varied immensely among countries in sub-Saharan Africa ranging from 1.8% in Zambia to 25.8% in Sierra Leone (Table 2). Similarly, the state of implementation of tobacco control policies varied across the region (Table 3). The highest smoking prevalences in SSA were reported in South Africa, Sierra Leone and Zambia. In general, smoking prevalences were higher in national and sub-national studies conducted in Malawi, Nigeria, Rwanda, Sierra Leone, and South Africa compared to prevalences in Ghana, Senegal, Togo and Uganda.

**Table 2:** Prevalence (95% CI) of current smoking among adults in sub-Saharan African countries by rural urban location or nationwide.

		BOTH MEN AND WOMEN Prevalence (95% CI)		
STUDY ID	COUNTRY	In Urban area	In Rural area	Overall nationwide prevalence
1	Congo	21.6	15.4	19.5
2	Ethiopia	**-**	-	-
3	Ghana	-	-	-
4	Ghana	-	-	(U) 3.4 (3.0, 3.9)
	Ghana	-	-	(A”) 3.8 (3.1, 4.4)
	Ghana	-	-	(A*) 4.3 (3.6, 5.0)
5	Kenya	4.09	-	-
	Kenya	1.24^✓^	-	-
6	Kenya	13.1	-	-
7	Kenya	-	6.3	-
	Kenya	-	5.7^π^	-
8	Malawi	6.6 (4.7, 8.5)	10.9 (10.0, 1.8)	14.1
9	Nigeria	(WP) 2.8 (2.4, 3.1)	(WP) 2.7 (2.4, 3.0)	(WP) 2.7
10	Nigeria	-	-	14.6
11	Rwanda	*18.2	*14.6	16.7
12	Senegal	-	-	5.8 (4.7, 7.2)
13	Sierra Leone	-	-	25.8 (23.4, 28.2)
14	South Africa	-	-	22
15	Togo	-	-	9.3
16	Uganda	-	2.2	-
17	Uganda	-	6.5 (5.8,7.1)	-
18	Uganda	-	-	6.4
19	Zambia	-	22.4	-
	Zambia	-	10.8	-
20	Zambia	-	-	1.8
21	Zambia	6.8	-	-

95% CI- 95% confidence interval; (U): Unadjusted

(A”): Adjusted for male under-response

(A*): Adjusted for male under-representation in the study sample, by population-based weighting using national survey data; (WP): weighted prevalence

*Not presented in paper, but enough data was presented for prevalence to be calculated manually.

π Tobacco smoking currently but not daily (within the last 30 days)

✓smoked daily;—Data not available from study

**Table 3:** Status of the implementation of selected articles under the supply and demand reduction measures, Treaty Provisions of the WHO FCTC.

		Supply reduction measures		Demand reduction measures				
		Sales to and by minors (Article 16)		Tobacco advertising promotion and sponsorship (Article 13)		Price and tax measures to reduce the demand of tobacco component of (Article 6)		
Country	Date of WHO FCTC ratification	Sale of tobacco products from vending machines prohibited	Sale of cigarettes individually or in small packets prohibited	Tobacco smoking banned in public places	Comprehensive ban on all tobacco, advertising promotion and sponsorship	Trends in prices	Tax policies to reduce tobacco consumption	Proportion of the retail price consisting of taxes
Congo	6 Feb 2007	**No**	**No**	Yes	Yes	Increased	Yes	32.0
Ethiopia	25 March 2014	No data	No data	No data	No data	No data	No data	No data
Ghana	29 November 2004	Yes	Yes	Yes	Yes	Constant for 2 years	Yes	88.0
Kenya	25 June 2004	Yes	Yes	Yes	Yes	Constant	Yes	52.0
Malawi	Not ratified	No data	No data	No data	No data	No data	No data	No data
Nigeria	28 June 2004	Yes	Yes	Yes	Yes	Increasing	Yes	Not answered
Rwanda	19 October 2005	Not answered	Not answered	Not answered	Not answered	No answer	Not answered	Not answered
Senegal	27 Jan 2005	No	No	Yes	Yes	Decline	Yes	70.9
Sierra Leone	22 May 2009	No	No	Yes	No	Not answered	No	Not answered
South Africa	19 April 2005	No	No	Yes	Yes	Decline	Yes	Not answered, 52.0 (2012)
Togo	15 Nov 2005	Yes	Yes	Yes	Yes	Stable	Yes	45.0
Uganda	20 June 2007	No	No	Yes	No	Stable for 2 years	Yes	40.3
Zambia	23 May 2008	No data	No data	No data	No data	No data	No data	No data

### Prevalence of current smoking by gender

The prevalence of smoking was consistently higher in men compared to women in all studies.[11, 12, 15, 16, 20, 22, 23, 26–28] Less than 5% of women reported currently smoking in all the studies included with the exception of Rwanda where more than 12% of women currently smoked.[27] This parallels an even higher prevalence of smoking among men (20.9%) in Rwanda.

### Comparison of current smoking prevalence between urban and rural locations

We obtained estimates of the prevalence of current smoking in both urban and rural areas within 8 countries; Congo,[29] Ghana,[11, 12] Ethiopia,[26] Kenya,[13, 19] Malawi,[15] Nigeria,[16] Rwanda[27] and Zambia.[24, 28] (Tables 2 and 4).

**Table 4:** Prevalence (95% CI) of current smoking by gender and urban and rural locations where available.

		MEN Prevalence (95% CI)			WOMEN Prevalence (95% CI)		
STUDY ID	COUNTRY	In Urban area	In Rural area	Overall prevalence	Overall prevalence	In Urban area	In Rural area
1	Congo	**-**	**-**	**-**	-	-	-
2	Ethiopia	10.3	21.6	18.3	1.0	0.7	1.1
3	Ghana	9.8	5.3	7.1	0.2	0.6	0.2
4	Ghana	3.4	3.1	8.9 (7.3, 10.5)	0.3 (0.1, 0.4)	0.22	0.13
5	Kenya	9.53	-	-	-	0.7	-
	Kenya	2.50^✓^	-	-	-	0.45^✓^	-
6	Kenya	22	-	-	-	3.8	-
7	Kenya	-	-	11.2	2.6	-	-
	Kenya	-	-	10.2^π^	2.3^π^	-	-
8	Malawi	-	-	25.9 (23.3, 28.5)	2.9 (2.1, 3.8)	-	-
9	Nigeria	-	-	(WP) 8.2 (7.6, 8.9)	(WP) 0.1 (0.1, 0.2)	-	-
10	Nigeria	-	-	-	-	-	-
11	Rwanda	*19.5	*22.8	20.9	12.6	*17	*7.0
12	Senegal	-	-	18.4	0.2	-	-
13	Sierra Leone	-	-	-	-	-	-
14	South Africa	-	-	-	-	-	-
15	Togo	-	-	20.2	3.0	-	-
16	Uganda	-	-	13.7	0.9	-	-
17	Uganda	13.1 (11.7,14.5)	-	-	-	-	1.3 (9.5,16.8)
18	Uganda	-	-	14.6	2.0	-	-
19	Zambia	-	39.6	-	-	-	10.8
	Zambia	-	40.4	-	-	-	7.2
20	Zambia	-	-	18.1	8.7	-	-
21	Zambia	17.5	-	-	-	1.5	-

95% CI- 95% confidence interval; (WP): weighted prevalence

*Not presented in paper, but enough data was presented for prevalence to be calculated manually.

π Tobacco smoking currently but not daily (within the last 30 days)

^✓^smoked daily;—Data not available from study

The greatest difference in current smoking prevalence between urban and rural areas was observed in Zambia. Smoking was 22.4% in rural Zambia,[21] compared to 6.8% in urban Zambia[24]. For Congo,[29] Ghana,[11, 12] Ethiopia,[26] Kenya,[13, 19] Malawi,[15] Nigeria,[16] Rwanda,[27] the difference in smoking between urban and rural areas were less marked. Rwanda and Zambia seemed to have the greatest variation in female smoking between urban and rural areas although with completely opposite patterns. In Zambia, more women smoked in rural areas compared to urban areas (10.8[28] vs 1.5[24]), yet in direct contrast a significantly higher proportion of Rwandan women smoked in urban locations (17 vs 7) than rural areas. The differences in smoking among men varied the most when men in urban and rural areas of residence for Ethiopia (21.6 rural vs 10.3 urban), Kenya (22 urban vs 11.2 rural) and Zambia (39.6 rural vs 17.5 urban) were compared.

### Consistency of findings and time trends

For five countries (Ghana, Kenya, Nigeria, Uganda and Zambia), at least two nationally representative population-based studies were conducted during 2007 to 2014. We observed variations in the smoking prevalences reported in studies conducted during the same year or time period and similar area e.g. a rural or urban area. For example, two studies conducted in urban areas in Kenya during 2010 estimated very different smoking prevalences 4%[30] and 13%.[19] Two very distinct rural smoking estimates were seen in rural Zambia as well (22.4% vs 10.8%).[21]

However, where cross-sectional studies have been conducted over successive years in the same type of area, both increases and declines in current self-reported smoking prevalence were observed. For Nigeria, a low prevalence of 2.7% in current smoking was reported in 2008[16] compared to 14.6% reported one year previously (2007).[25] the smoking prevalence previously reported in rural Uganda during 2008–2009 (2.2%)[31] increased slightly to 6% in 2011.[17]

### Tobacco control strategies present among countries

All countries included in the review except for Malawi are a signatory to the treaty and have ratified or obtained accession to the WHO’s Framework Convention on Tobacco Control.[32] Ethiopia took the longest time (10 years) to ratify the convention after becoming a signatory. All other countries took less than 3 years to formally approve the treaty after becoming signatories. Among the 13 countries, according to the results from countries’ status of the implementation of the policies under the WHO FCTC, only policies that are considered to be complete and not in draft form were reported on. Data were available for 9 countries (Congo, Ghana, Kenya, Nigeria, Senegal, Sierra Leone, South Africa, Togo, and Uganda) either because of the lack of the country’s authorities submitting a report or not answering a particular survey question (Table 3). Of these countries, 7 had comprehensive bans on all tobacco advertising promotion and sponsorship, but Sierra Leone and Uganda did not. All 9 countries had implemented bans on smoking tobacco in public places. All except for Sierra Leone had some taxation policies in place to reduce tobacco consumption. We noticed more variation in the percentage tax on the most popular price category of tobacco product among the countries. As of 2014 according to results from the survey, most of the countries for which data was available had set tobacco taxes below 70% except for Ghana (88%) and Senegal (70.9%). Recent trends in cigarette price have been increasing in Congo and Nigeria, but have declined in Senegal and South Africa. However they have been constant in Ghana, Kenya, Togo and Uganda for approximately 2 years.

## Discussion

### Summary of main findings on current smoking prevalence

We observed a wide range of current smoking prevalence among the 13 sub-Saharan African countries in the review. This highlights the presence of wide gender diversity in smoking prevalence that is much more prominent in sub-Saharan Africa and other developing countries than in developed countries.[1] The low observed prevalence of smoking among women in sub-Saharan Africa may be due to the presence of strong social norms and taboos which discourage women to smoke.[33, 34] In the same way these social norms may depict smoking among women as inappropriate, smoking among men in some societies is viewed as acceptable, and as a symbol of status and social power. There may also be a growing parity between the prevalence of smoking among men in a few sub-Saharan African countries and those in Western societies reflecting global patterns which shows an increasing similarity in the prevalence of smoking among men in low, middle and high income regions of the world.[35] The prevalence of smoking differed according to location of residence among adults in the same country. We observed that more adults residing in urban areas smoked compared to rural areas for some countries. This may be indicative that factors related to urbanization, including economic factors, are having an impact on individuals’ ability to have greater access to cigarettes in some African societies.[35] However, we do not know what the underlying characteristics of the urban residents are, for example, whether it is due to higher socioeconomic status which may explain the higher smoking prevalence observed, or simply greater access. Some studies reported diverse prevalence ranges for the same geographical area i.e. rural or urban, which might even illustrate different smoking norms are present among different areas within a country.

The differences in reported smoking prevalence may be a reflection of the different sampling strategies used in the different studies. The differences in smoking observed between studies in Nigeria may be due to differences in the age groups and sample size of the populations studied. The 14.6% prevalence was from a slightly older population aged 25–64 using a sample selected from one region in Nigeria, not using a nation-wide sampling frame and also using a much smaller sample size (1458) compared to the younger 15–59 population where the 2.7% prevalence was observed from a sample comprising 48871 participants. It is possible that the higher prevalence observed in Nigeria may be specific to that region and less representative of the wider Nigerian population. It may also be due to inconsistencies in measuring current smoking which we realized varied greatly across studies. Our attempts to compare the different smoking prevalences are limited by the different sampling procedures and methods of measuring current smoking.

### Comparison to previous findings

**Fig 2:**
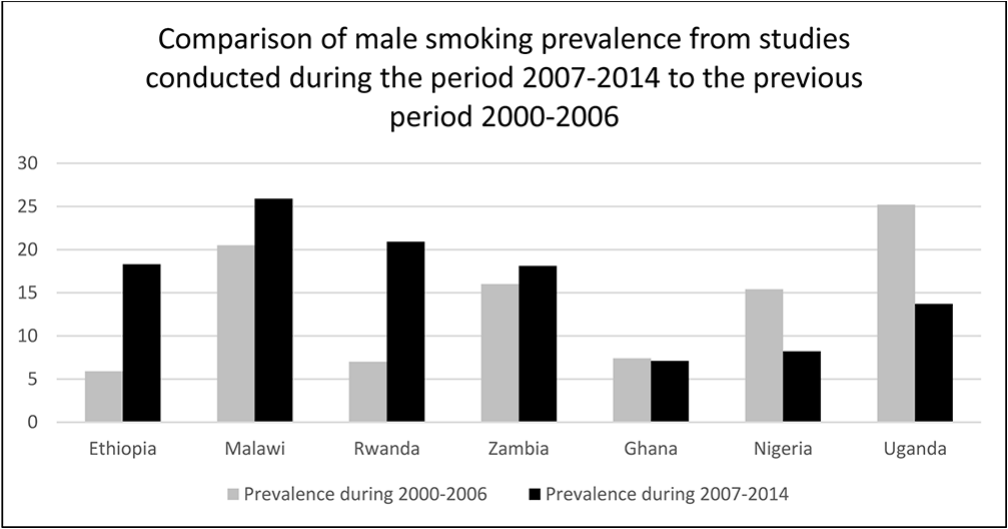
Reported smoking prevalence during 2000–2006 and 2007–2014 for men in 7 countries in sub-Saharan Africa that were included in the review.

**Fig 3:**
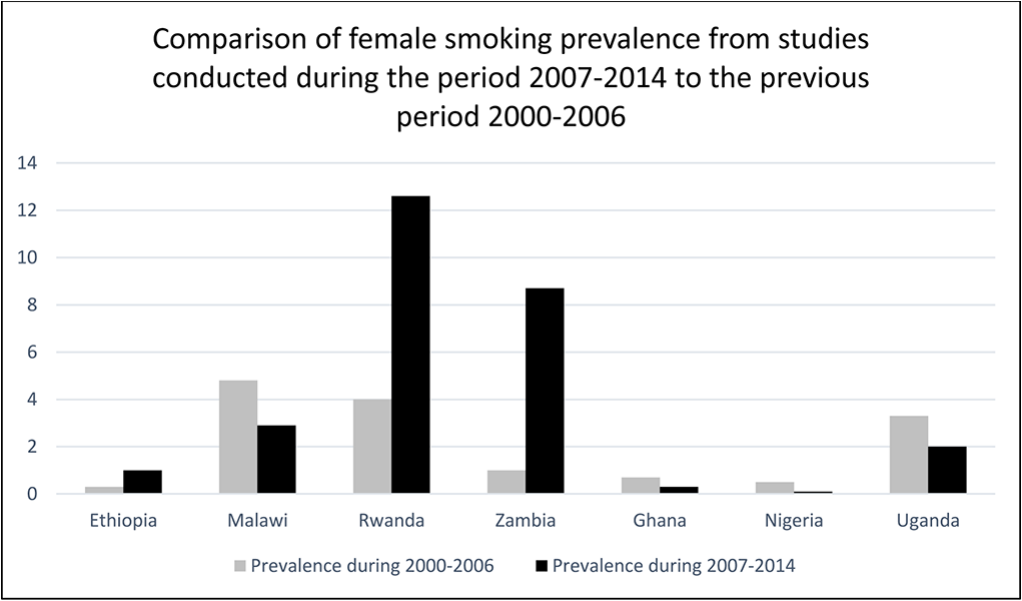
Reported smoking prevalence during 2000–2006 and 2007–2014 for women in 7 countries in sub-Saharan Africa that were included in the review.

We compared our findings to a systematic review for the period 2000–2006.[36] In comparing the current review period (2007–2014) to the previous review period, we looked for studies that reported overall prevalence of smoking for an area representative of the entire country and not just a rural or urban area and separately by gender. Five countries also had an estimate reported in the previous period of 2000–2006. The comparisons are shown in Figs 2 and 3.

The patterns observed from comparing smoking measured during the periods 2000–06 and 2007–14 varied quite markedly by country and by gender. Estimated smoking prevalence was higher in the most recent review among both men and women in Ethiopia, Rwanda and Zambia (Figs 2 and 3). In contrast, smoking prevalence was lower in studies in the recent review among both men and women in Ghana, Nigeria and Uganda. In Kenya smoking prevalence among women was higher but among men was lower in the latest review while the opposite pattern was seen in Malawi. These descriptive patterns are interesting but we did not conduct further analysis as different methodologies were used in the two periods and the populations studied also differed.

### Description of the tobacco control measures present in 13 sub-Saharan African countries

The tobacco industry has been successful in expanding its markets to low and middle-income countries by capitalising on economic growth, changing social norms and population demographics in low income regions.[1] Africa has lower rates of tobacco taxation, weaker smoke-free policies and less stringent tobacco advertising restrictions in comparison to higher income countries.[9, 37] Tobacco companies are known to be attracted to weak policy environments and execute stronger tactics which oppose governments’ fight for smoke free environments.[38] From the included studies, very high prevalences of smoking among men was observed in Malawi (25.9%) who is yet to ratify the WHO FCTC convention, and in rural Ethiopia (21.6%) who has ratified the convention very late in 2014, since it was created under the WHO in response to the global tobacco epidemic as a strategy to reduce the demand and supply of tobacco in countries. Malawi’s adoption of the WHO FCTC would help in implementing methods to regulate the demand and supply of tobacco which will involve both economic and non-economic measures to be executed and help reduce the high smoking rates among men in the country. Further to this, we noticed that Ghana, Nigeria, Togo and Senegal adopted the WHO FCTC earlier and from the data we observed that they have lower smoking rates than countries who signed the convention much later such as Sierra Leone. There can be many explanations for such a relationship, but earlier adoption of the WHO FCTC can allow governments the structure to be able to enforce stronger tobacco control policy environments.

Advertising is known to increase tobacco consumption by increasing sales and may even trigger potential users to start smoking.[39] Research conducted among 22 Organization for Economic Cooperation and Development (OECD) countries concluded that the presence of comprehensive bans are better able to reduce consumption than limited bans. In the absence of comprehensive bans in Sierra Leone and Uganda, a considerable proportion of men smoked in Uganda (13.7%) including rural areas (14.0%) and among adults in the population of Sierra Leone (25.8%) have been documented. The absence of robust tobacco control policies might be influencing the high smoking rates in these countries. However, this is not a simple relationship. There is likely to be a wide range of factors influencing whether adults smoke in sub-Saharan Africa, which include cultural and religious factors that determine the social acceptability of smoking, and the effectiveness of the implementation of tobacco control polices.

With respect to taxes, the literature has shown that tobacco taxes when raised to increase prices can reduce and stop consumption among current users and or prevent consumption among potential users, significantly impacting young persons and poorer users.[40, 41] It is recommended that tobacco excise taxes be set above 70% of the retail price of the tobacco product to have a significant impact on increasing prices and thereby reducing consumption. The data reported here do not allow us to draw any conclusions about the effectiveness of control measures. For example, a government concerned about high levels of existing smoking might introduce stricter controls whereas in areas with lower smoking, tobacco policies may receive less attention. The one clear conclusion is that tobacco control policies vary widely in different African countries, with no clear correlation with smoking prevalence.

### Strengths and limitations

We believe that the included studies give good estimates of current smoking prevalence in the respective countries, and rural or urban sub-national areas over the last seven years (2007 to May 2014). We were unable to make any analysis of the association between preventive measures and smoking prevalence due to several methodological and data limitations. Most importantly is that the date the prevalence of current smoking was measured may precede the implementation of the respective tobacco control measures. We know that the measures would have been implemented as of 2014 but some of the prevalences reported may not have been impacted by the presence of these tobacco control measures if the measures were implemented after the study was done. Any possible associations are subject to a number of other confounding factors at the individual and community level.

This review may also be limited in the strength of comparisons made between the prevalence of smoking between urban and rural areas and across countries, since the research varied in how smoking was measured, how representative samples were selected, and the period in which the data were collected. We only looked at data within the broad period 2007 to 2012 and not specifically by year, so we are unable to deduce whether an increasing or decreasing trend in current smoking was present.

Another bias amongst the studies may be in the measurement of current smoking. All included studies assessed current smoking from self-reported statuses. Past research observed underreporting among patients who had their current smoking status confirmed via taking measurements of their carbon monoxide level and concentration of serum cotinine. Approximately 50% of current smokers self-reported to be non-smokers.[42] Furthermore, smoking in certain countries in Africa seems to be stigmatised especially among women.[43] There may be a possibility that the prevalence may be underreported for example in some countries with unusually low prevalence such as Ghana.

## Implications for Policy

In spite of the limitations of the data, this study has important implications for public health research and policy in Africa. It shows that smoking levels are still high in the majority of sub-Saharan African countries for which data are available. There is also some evidence to suggest smoking prevalence may be increasing in some areas, especially among women. In addition, we found that the level of implementation of tobacco control measures varies widely for the countries in the review, although due to data limitations we could not make any analysis of the effect of prevention policy, not the effects of policy gaps. Health policy makers would benefit from more reliable and complete data on smoking prevalence trends for all countries in sub-Saharan Africa including within country patterns of smoking by age, gender and ideally comparing rural-urban environments. Data on smoking prevalence and also better evidence of the effectiveness of tobacco control policies in Africa are needed to demonstrate that smoking should, and can, be addressed as a major population health priority in Africa. The continued influx of international aid for infectious diseases, such as malaria and HIV/AIDS, has caused distortions within health systems and health policy priorities, and continues to draw resources away from tackling non-communicable disease, and the single biggest risk factor, smoking.

## Conclusion

This shows that smoking in some countries of sub-Saharan Africa is increasing. The patterns varied across the region, within sub-regions and by rural urban location within countries. Gender seems to be the strongest determinant of tobacco smoking among adults, with men smoking more than women in all countries. The implementation of tobacco control policies was also found to vary widely, in the study countries. More research is needed on the implementation and effectiveness of tobacco control policies across all countries in sub-Saharan Africa. Effective action to reduce tobacco smoking and to particularly stop the increased uptake of smoking among women must become a public health priority for sub-Saharan Africa to reduce the burden of disease.

## Supporting Information

S1 Checklist PRISMA 2009 Checklist. (DOC)

S1 Appendix Example search strategy. (DOCX)

S2 Appendix Reasons for excluding studies from systematic review. (DOCX)

S3 Appendix Details of the sampling methodology and measurement of current smoking used in each study. (DOCX)
