# Correction: A Systematic Review of Tobacco Smoking Prevalence and Description of Tobacco Control Strategies in Sub-Saharan African Countries; 2007 to 2014

**DOI:** 10.1371/journal.pone.0155572

**Published:** 2016-05-09

**Authors:** The PLOS ONE Staff

There is an error regarding copyright. The license is incorrect. The correct copyright statement should read: This is an open access article distributed under the terms of the Creative Commons Attribution License, which permits unrestricted use, distribution, and reproduction in any medium, provided the original author and source are credited. The publisher apologizes for the error.
