# Supplementary material for: A Systematic Review of Tobacco Smoking Prevalence and Description of Tobacco Control Strategies in Sub-Saharan African Countries; 2007 to 2014
Source: PLoS One. 2015 Jul 10;10(7):e0132401. doi: 10.1371/journal.pone.0132401 (PMC4498629; doi:10.1371/journal.pone.0132401)
Supplement: S1 Appendix — (DOCX) [file pone.0132401.s002.docx]

**Appendix S1. A systematic review of tobacco smoking prevalence and description of tobacco control strategies in sub-Saharan African countries; 2007 to 2014.**

**Web-only Appendix S1:** **Example search strategy**

The search strategy involved a combination of three different concepts:

1. Sub-Saharan Africa **AND**
2. Smoking **AND**
3. Types of studies

The following search strategy was used in most of the databases with slight adaptations for databases using different interfaces and thus required the use of different subject headings.

**Table 1. Example Search strategy used in the OvidSP databases**

| 1 | exp “Africa south of the Sahara”/ |
| --- | --- |
| 2 | sub-Saharan Africa |
| 3 | subsaharan Africa |
| 4 | Black Africa |
| 5 | Africa south of the Sahara |
| 6 | Exp “Democratic Republic of the Congo”/ |
| 7 | Angola or Benin or Botswana or Burkina Faso or Burundi or Cameroon or Cape Verde or Central African Republic or CHAD or Comoros or Congo or Congo Democratic Republic or Djibouti or Equatorial Guinea or Eritrea or Ethiopia or Gabon or Gambia or Ghana or Guinea or Guinea-Bissau or Cote d'Ivoire or Ivory Coast or Kenya or Lesotho or Liberia or Madagascar or Malawi or Mali or Mozambique or Namibia or Niger or Nigeria or (Sao tome and Principe) or Rwanda or Senegal or Seychelles or Sierra Leone or Somalia or South Africa or South Sudan or Sudan or Swaziland or Tanzania or Togo or Uganda or Zambia or Zimbabwe |
| **8** | **1 -7** |
|  | **Search terms for smoking** |
| 9 | Smoking |
| 10 | Smok* or tobacco* or cigar* |
| 11 | Tobacco consumption |
| 12 | Tobacco/ |
| 13 | Pipe adj2 smok* |
| 14 | Bidi |
| 15 | Kretek |
| 16 | prevalence ADJ4 tobacco |
| 17 | prevalence ADJ4 smoking |
| **18** | **9 or 10 or 11 or 12 or 13 or 14 or 15 or 16 or 17** |
|  | **Search terms for types of studies** |
|  | **Search filter for type of studies that may have prevalence** |
| 19 | Exp Prevalence/ |
| 20 | Prevalence |
|  | **Search filter for cross-sectional studies (Ovid Medline)** |
| 21 | exp Cross-Sectional Studies/ or cross-sectional.ti,ab. or "prevalence study".ti,ab. |
|  | **Search filter for cohort studies (Ovid Medline)** |
| 22 | cohort.ti,ab. or exp Cohort Studies/ or longitudinal.ti,ab. or prospective.ti,ab. or retrospective.ti,ab. |
|  | **Search filter for epidemiologic studies (Ovid Medline)** |
| 23 | Epidemiologic Studies/ |
|  | **Combining the searches** |
| **24** | **19 or 20 or 21 or 22 or 23** |
| **24** | **8 AND 18 AND 24** |
| **25** | **Limit 24 to the studies published from the years 2007 to 2014 (May)** |
