# Supplementary material for: A Systematic Review of Tobacco Smoking Prevalence and Description of Tobacco Control Strategies in Sub-Saharan African Countries; 2007 to 2014
Source: PLoS One. 2015 Jul 10;10(7):e0132401. doi: 10.1371/journal.pone.0132401 (PMC4498629; doi:10.1371/journal.pone.0132401)
Supplement: S2 Appendix — (DOCX) [file pone.0132401.s003.docx]

**Appendix S2. A systematic review of tobacco smoking prevalence and description of tobacco control strategies in sub-Saharan African countries; 2007 to 2014.**

**Web-only Appendix S2-Reasons for excluding studies from systematic review**

Table 1 showing reasons why articles were excluded from the systematic review

| **Reference** | **Reason for exclusion** |
| --- | --- |
| ^1^ | Prevalence of current smoking not reported |
| ^2^ | Prevalence of current smoking not reported in representative sample |
| ^3^ | Current smoking prevalence not reported  Small sample size less than 1000 participants- 805 |
| ^4^ | Current smoking prevalence not reported  Community based-Small sample size less than 1000 participants, 444 |
| ^5^ | Prevalence of current smoking combined with ex- smoking |
| ^6^ | Prevalence of current smoking not reported, only ever smoking and use of chewing tobacco and snuff |
| ^7^ | Prevalence of current smoking not reported |
| ^8^ | Prevalence of current smoking not reported, Data collection period before 2007 (2005) |
| ^9^ | Prevalence of current smoking not reported |
| ^10^ | Small sample size (419) longitudinal study, no prevalence of current smoking reported |
| ^11^ | No prevalence of current smoking |
| ^12^ | Unrepresentative community-based sample  21.5% response rate (207 participants) |
| ^13^ | Community-based  Small sample size 407 participants |
| ^14^ | Community based  Sample size less than 1000- 611 participants |
| ^15^ | The majority of the sample represented adults over 50 years (4770) and a small number between ages 18-49 (803) |
| ^16^ | Community based- sample sizes of urban and rural areas less than 1000 |
| ^17^ | Not in sub-Saharan Africa |
| ^18^ | Adults over 40 years |
| ^19^ | Semi-urban area |
| ^20^ | Aged 35 and above |
| ^21^ | Unrepresentative |
| ^22^ | Unrepresentative community-based survey |
| ^23^ | Unrepresentative community-based survey |
| ^24^ | Aged 51 and older |
| ^25^ | Aged 50 and older |
| ^26^ | Unrepresentative community-based survey |
| ^27^ | Unrepresentative community-based survey |
| ^28^ | Unrepresentative community-based survey |
| ^29^ | Data collection period before 2007, (2004,2005) |
| ^30^ | Data collected before 2007, (2003) |
| ^31^ | Data collected before 2007 (2004) |
| ^32^ | Data collected before 2007 (2006) |
| ^33^ | Data collected before 2007 (2006) |
| ^34^ | Data collection before 2007- (2002-2004) |
| ^35^ | Data collection period before 2007 (2005) |
| ^36^ | Data collection period before 2007 (2005) |
| ^37^ | Data collection period before 2007 (2006) |
| ^38^ | Data collection period before 2007 (2006) |
| ^39^ | Data collection period before 2007 (2002-2004) |
| ^40^ | Data collection period before 2007 (1996-1998) |
| ^41^ | Data collected before 2007 (2002-2003) |
| ^42^ | Sample size less than 1000- 548 |
| ^43^ | Study sample less than 1000 participants (658) |
| ^44^ | Sample size less than 1000 (469) |
| ^45^ | Sample size less than 1000 (699) |
| ^46^ | Sample size less than 1000- (899) |
| ^47^ | Sample size less than 1000 (200) |
| ^48^ | Sample size less than 1000 participants |
| ^49^ | Sample size less than 1000 participants (886) |
| ^50^ | Data collection period before 2007 (2005-2006) |
| ^51^ | Study period before 2007 (2004) |
| ^52^ | Data collection period before 2007 (2002-2005) |
| ^53^ | Data collection period before 2007 (2005) |
| ^54^ | Data collection period before 2007 (2003) |
| ^55^ | Data collection period before 2007 (2002), sample size less than 1000 (200) |
| ^56^ | No full text |
| ^57^ | Period of data collection not reported |
| ^58^ | Review |

**References**

1. Agyemang C, Owusu-Dabo E. Prehypertension in the Ashanti region of Ghana, West Africa: an opportunity for early prevention of clinical hypertension. Public Health 2008;**122**(1):19-24.

2. Bovet P, Gervasoni JP, Mkamba M, et al. Low utilization of health care services following screening for hypertension in Dar es Salaam (Tanzania): A prospective population-based study. BMC Public Health 2008;**8**(407).

3. Desalu OO, Salami AK, Oluboyo PO. Self-reported risk factors of asthma in a nigerian adult population. Turk Toraks Dergisi 2009;**10**(2):56-62.

4. Ekanem US, Opara DC, Akwaowo CD. High blood pressure in a semi-urban community in south-south Nigeria: a community-based study. Afr Health Sci 2013;**13**(1):56-61.

5. Okpechi IG, Chukwuonye II, Tiffin N, et al. Blood pressure gradients and cardiovascular risk factors in urban and rural populations in Abia State south eastern Nigeria using the WHO STEPwise approach. PLoS ONE 2013;**8**(9).

6. Onwuchekwa AC, Mezie-Okoye MM, Babatunde S. Prevalence of hypertension in Kegbara-Dere, a rural community in the Niger Delta Region, Nigeria. Ethn Dis 2012;**22**(3):340-46.

7. Onwuchekwa AC, Tobin-West C, Babatunde S. Prevalence and risk factors for stroke in an adult population in a rural community in the Niger Delta, South-South Nigeria. J Stroke Cerebrovasc Dis 2014;**23**(3):505-10.

8. Pisa PT, Kruger A, Vorster HH, et al. Alcohol consumption and cardiovascular disease risk in an African population in transition: the Prospective Urban and Rural Epidemiology (PURE) study. (Special Issue: Alcohol consumption in South Africa: from molecules to society.). SAJCN South African Journal of Clinical Nutrition 2010;**23**(3, Suppl. 1):S29-S37.

9. Rudatsikira E, Muula AS, Mulenga D, et al. Prevalence and correlates of obesity among Lusaka residents, Zambia: A population-based survey. Int Arch Med 2012;**5**(1).

10. Sossa C, Delisle H, lène, et al. Lifestyle and dietary factors associated with the evolution of cardiometabolic risk over four years in West-African adults: the Benin study. Journal of obesity 2013.

11. Kasiam LO, Longo-Mbenza B, Nge OA, et al. Classification and dramatic epidemic of diabetes mellitus in Kinshasa Hinterland: the prominent role of type 2 diabetes and lifestyle changes among Africans. Nigerian journal of medicine : journal of the National Association of Resident Doctors of Nigeria 2009;**18**(3):311-20.

12. Ahmed HG. Survey on knowledge and attitudes related to the relation between tobacco, alcohol abuse and cancer in the northern state of Sudan. Asian Pac J Cancer Prev 2013.

13. Desalu OO, Salami AK, Fawibe AE. Prevalence of cough among adults in an urban community in Nigeria. West Afr J Med 2011;**30**(5):337-41.

14. Mondo CK, Otim MA, Akol G, et al. The prevalence and distribution of non-communicable diseases and their risk factors in Kasese district, Uganda. Cardiovascular Journal of Africa 2013;**24**(3):52-7.

15. Saeed BII, Abdul-Aziz AR, Nguah SB, et al. The impact of Preventive Health Behaviors and Risk Factors on Health Status of Ghanaians. Global journal of health science 2013.

16. Zyl Sv, Merwe LJvd, Walsh CM, et al. Risk-factor profiles for chronic diseases of lifestyle and metabolic syndrome in an urban and rural setting in South Africa. African Journal of Primary Health Care and Family Medicine 2012;**4**(1).

17. Blecher E, Liber AC, Chaussard M, et al. Market structures, socioeconomics, and tobacco usage patterns in Madagascar. Nicotine Tob Res 2014;**16**(SUPPLEMENT1):S56-S64.

18. Brian G, Ramke J, Szetu J, et al. Cataract and its surgery in Fiji. Clin Experiment Ophthalmol 2011;**39**(5):449-55.

19. Dahiru T, Ejembi CL. Clustering of cardiovascular disease risk-factors in semi-urban population in Northern Nigeria. Nigerian Journal of Clinical Practice 2013;**16**(4):511-6.

20. Desalu OO. Prevalence of chronic bronchitis and tobacco smoking in some rural communities in Ekiti state, Nigeria. The Nigerian postgraduate medical journal 2011;**18**(2):91-97.

21. Diouf M, Boetsch G, Tal-Dia A, et al. Digestive pathology and oral condition in the rural populations of the Ferlo in Senegal. Pathologie digestive et é;tat bucco-dentaire chez les populations rurales du Ferlo au Sé;né;gal 2013.

22. Ekwom PE, Oyoo GO, Ongore D. Prevalence of musculoskeletal pain in Nairobi, Kenya: Results of aphase 1, Stage 1 COPCORD study. Clin Rheumatol 2013;**1)**:S121.

23. Pires J, Sebastiao Y, Rodrigues A, et al. Hypertension prevalence in northern angola: Current status and future challenges. J Hypertens 2012;**30**:e134-e35.

24. Ploubidis GB, Mathenge W, De Stavola B, et al. Socioeconomic position and later life prevalence of hypertension, diabetes and visual impairment in Nakuru, Kenya. Int J Public Health 2013.

25. Yawson AE, Baddoo A, Hagan-Seneadza NA, et al. Tobacco use in older adults in Ghana: sociodemographic characteristics, health risks and subjective wellbeing. BMC Public Health 2013;**13**:979.

26. Ogola EN, Ahmed AH, Joshi MD, et al. Prevalence of hypertension and cardiovascular risk factors in ethnic somali residents of north eastern kenya. J Hypertens 2012;**30**:e135.

27. Musinguzi G, Nuwaha F. Prevalence, awareness and control of hypertension in Uganda. PLoS ONE 2013.

28. Ogah OS, Madukwe OO, Onyeonoro UU, et al. Cardiovascular risk factors and non-communicable diseases in Abia state, Nigeria: report of a community-based survey. International Journal of Medicine and Biomedical Research 2013;**2**(1):57-68.

29. Dutra LM, Williams DR, Gupta J, et al. Human rights violations and smoking status among South African adults enrolled in the South Africa Stress and Health (SASH) study. Soc Sci Med 2014;**105**:103-11.

30. Joseph M, Rachel J, Nicola S, et al. Prevalence of Alcohol Consumption and Hazardous Drinking, Tobacco and Drug Use in Urban Tanzania, and Their Associated Risk Factors. Int J Environ Res Public Health 2009:1991-2006.

31. Kengne AP, Awah PK, Fezeu L, et al. The burden of high blood pressure and related risk factors in urban Sub-Saharan Africa: Evidences from Douala in Cameroon. Afr Health Sci 2007;**7**(1):38-44.

32. Masalu JR, Kikwilu EN, Kahabuka FK, et al. Oral health related behaviors among adult Tanzanians: a national pathfinder survey. BMC Oral Health 2009;**9**(22).

33. Meseret A, Bejiga A, Ayalew M. Prevalence of Pterygium in a Rural Community of District, Southern Ethiopia. The Ethiopian Journal of Health Development 2008;**22**(2):191-94.

34. Myers B, van Heerden MS, Grimsrud A, et al. Prevalence and correlates of atypical patterns of drug use progression: Findings from the South African Stress and Health Study. African Journal of Psychiatry (South Africa) 2011;**14**(1):38-44.

35. On'Kin JBKL, Longo-Mbenza B, Okwe N, et al. Prevalence and risk factors of diabetes mellitus in Kinshasa Hinterland. International Journal of Diabetes and Metabolism 2008;**16**(3):97-106.

36. Sebhatu M, Kiflom B, Seyoum M, et al. Determining the burden of tuberculosis in Eritrea: A new approach. Bull World Health Organ 2007;**85**(8):593-99.

37. Tesfaye F, Byass P, Wall S. Population based prevalence of high blood pressure among adults in Addis Ababa: uncovering a silent epidemic. BMC Cardiovasc Disord 2009.

38. Tesfaye F, Byass P, Wall S, et al. Association of smoking and khat (Catha edulis Forsk) use with high blood pressure among adults in Addis Ababa, Ethiopia, 2006. Prev Chronic Dis 2008;**5**(3):A89.

39. van Heerden MS, Grimsrud AT, Seedat S, et al. Patterns of substance use in South Africa: Results from the South African Stress and Health study. S Afr Med J 2009;**99**(5):358-66.

40. Vorster HH, Kruger A, Venter CS, et al. Cardiovascular disease risk factors and socio-economic position of Africans in transition: The THUSA study. Cardiovascular Journal of Africa 2007;**18**(5):282-89.

41. Gureje O, Degenhardt L, Olley B, et al. A descriptive epidemiology of substance use and substance use disorders in Nigeria during the early 21st century. Drug Alcohol Depend 2007;**91**(1):1-9.

42. Reda AA, Kotz D, Biadgilign S. Adult tobacco use practice and its correlates in eastern Ethiopia: A cross-sectional study. Harm Reduct J 2013;**10**(1).

43. van Zyl S, van der Merwe LJ, Walsh CM, et al. A risk-factor profile for chronic lifestyle diseases in three rural Free State towns. South African Family Practice 2010;**52**(1):72-76.

44. Jenson A, Omar AL, Omar MA, et al. Assessment of hypertension control in a district of Mombasa, Kenya. Glob Public Health 2011;**6**(3):293-306.

45. Katchunga PB, Masumbuko BE, Lemogoum D, et al. Hypertension in the adult Congolese population of Southern Kivu: Results of the Vitaraa Study. [French]

Hypertension arterielle chez l'adulte Congolais du Sud Kivu: Resultats de l'etude Vitaraa. Presse Med 2011;**40**(6):e315-e23.

46. Mbatia J, Jenkins R, Singleton N, et al. Prevalence of alcohol consumption and hazardous drinking, tobacco and drug use in urban Tanzania, and their associated risk factors. Int J Environ Res Public Health 2009;**6**(7):1991-2006.

47. Sodjinou R, Agueh V, Fayomi B, et al. Obesity and cardio-metabolic risk factors in urban adults of Benin: Relationship with socio-economic status, urbanisation, and lifestyle patterns. BMC Public Health 2008;**8**(84).

48. M'Buyamba-Kabangu J, Lemogoum D, Bayauli P, et al. The burden of cardiovascular risk factors among the inhabitants of an urban congolese community: Results of the vitaraa study. J Hypertens 2010;**28**:e137.

49. Zyl Sv, Merwe LJvd, Walsh CM, et al. A risk-factor profile for chronic lifestyle diseases in three rural Free State towns: original research. South African Family Practice 2010;**52**(1):72-76.

50. Kouassi B, Kpebo OD, Horo K, et al. Smoking and educational status in Africans. Tabagisme et niveau d'instruction en milieu africain 2010.

51. Mufunda J, Debesay A, Mosazghi A, et al. Prevalence of tobacco use in Eritrea: Results from a noncommunicable disease risk factor survey. Nicotine Tob Res 2007;**9**(7):777-79.

52. Oladapo OO, Salako L, Sodiq O, et al. A prevalence of cardiometabolic risk factors among a rural Yoruba south-western Nigerian population: A population-based survey. Cardiovascular Journal of Africa 2010;**21**(1):26-31.

53. Padrao P, Damasceno A, Silva-Matos C, et al. Tobacco consumption in Mozambique: Use of distinct types of tobacco across urban and rural settings. Nicotine Tob Res 2013;**15**(1):199-205.

54. Padrao P, Silva-Matos C, Damasceno A, et al. Association between tobacco consumption and alcohol, vegetable and fruit intake across urban and rural areas in Mozambique. J Epidemiol Community Health 2011;**65**(5):445-53.

55. Thorogood M, Connor M, Tollman S, et al. A cross-sectional study of vascular risk factors in a rural South African population: Data from the Southern African Stroke Prevention Initiative (SASPI). BMC Public Health 2007;**7**(326).

56. Lemogoum D, Bayauli P, Toto Moukouo J, et al. Gender difference in the burden of cardiovascular risk factors among rural and urban adult cameroonian populations. Results from the vitaraa study. J Hypertens 2010;**28**:e134-e35.

57. Andy JJ, Peters EJ, Ekrikpo UE, et al. Prevalence and correlates of hypertension among the Ibibio/Annangs, Efiks and Obolos: A cross sectional community survey in rural South-South Nigeria. Ethn Dis 2012;**22**(3):335-39.

58. Silva-Matos C, Beran D. Non-communicable diseases in Mozambique: risk factors, burden, response and outcomes to date. Globalization and Health 2012;**8**(37).
