# Supplementary material for: A Systematic Review of Tobacco Smoking Prevalence and Description of Tobacco Control Strategies in Sub-Saharan African Countries; 2007 to 2014
Source: PLoS One. 2015 Jul 10;10(7):e0132401. doi: 10.1371/journal.pone.0132401 (PMC4498629; doi:10.1371/journal.pone.0132401)
Supplement: S3 Appendix — (DOCX) [file pone.0132401.s004.docx]

**Appendix S3. A systematic review of tobacco smoking prevalence and description of tobacco control policies in sub-Saharan African countries; 2007 to 2014.**

**Web-only Appendix S3- Details of the sampling methodology and measurement of current smoking used in each study**

**Table 1 Details of the sampling methodology and measurement of current smoking used in each study**

| **Study ID** | **Country/ area representative of** | **Sampling strategy** | **Actual sample size** | **How smoking was defined in the study** |
| --- | --- | --- | --- | --- |
| 1 | Congo, Kinshasa sub-national, rural and urban | Households were sampled in four stages. In the first stage, eight health zones were randomly selected from the 35 health zones in Kinshasa. The second stage involved the random selection of 2 health areas in each health zone. The third stage randomly chose three streets from among all the listed streets within each health area. Fourthly, inhabited parcels were randomly selected from the list of whole parcels. One household was randomly chosen in instances where more than one household was present on a parcel.  Individuals met the inclusion criteria if they were older than 5 years and lived in Kinshasa for more than 1 year.  One individual was randomly recruited to participate from among those who met the inclusion criteria in the household. The number of participants was selected proportionately according to the size of the health zone. | 1412 | Active smoking |
| 2 | Ethiopia, sub-national, rural and urban | Residents aged 15 to 64 years living in the 10 selected administrative areas under research in Ethiopia. The population was stratified by sex, age and rural urban residential area | 5500 | Current smoker  Yes/No |
| 3 | Ghana, national, urban and rural | The Demographic and Health Survey conducted in 2008 in Ghana sampled households which were representative of Ghana. This was done using a two-stage cluster design. 11913 households were given a general questionnaire and half of the households were given additional questions for men and women. | Total 9484  4916 men  4568 women | Smoking was defined in 2 ways:   1. Currently consumed cigarettes 2. Currently consumed cigarettes, cigars, pipe tobacco, chewing tobacco, and or snuff |
| 4 | Ghana, sub-national urban and rural | The study was conducted in the Ashanti region of Ghana which is located in the central area of Ghana and is the most densely populated among the 10 regions in Ghana and most representative of the national population. All Ashanti enumeration areas listed in the 2000 census comprised the sampling frame. The list was stratified according to urban rural location and a random sample of 15 EAs was selected from each area. Field workers from Ghana Statistical office visited each enumeration area to select 20% of the houses in the area for the sample. An enumeration area typically contains approximately 100 houses, the field workers marked with chalk every fifth house as they walked the route.  Institutionalised individuals were excluded | 6258 | Smokes at least 100 cigarettes in his or her lifetime and smokes nowadays |
| 5 | Kenya/ sub-national- peri-urban | The study was conducted in the Wemuye HDSS site. The area was stratified using random sampling by sub-location and village using probability proportional to size. Individuals were randomly selected from male and female clusters in each sub-location and village. | 4037 | Tobacco used currently daily- within the last 30 days |
| 6 | Kenya, sub-national Urban slum of Nairobi (the capital of Kenya) | Urban slum in Nairobi Kenya called Kibera. The sample was drawn from a list of villages in this area. Clusters were sampled based on probability proportional to size of the village. The design effect of 2 was used to adjust for the degree of clustering among the participants. 80 clusters each containing about 10 households were selected. 25 participants per cluster were selected. The nearest health center, church or school was used as the starting point from which recruitment of households began. Households were selected through a random walk from the starting point. The next consecutive household was also selected from the recruited ones until the sample size was reached. All adults over 18 years living in the area for more than 3 months were invited to participate in the study. All pregnant women were excluded. Two visits were made to each household in an attempt to recruit residents absent at the time of first visit. | 2061 | Current cigarette smokers |
| 7 | Kenya/sub-national rural western Kenya | The study was conducted in the Rarieda, Wagai, Yala and Karemo District of rural western Kenya. The Health and Demographic Surveillance System is a longitudinal based database of demographic and health census data. Household interviews were conducted in during the second round of the HDSS survey. Senior adult members of the household were used as proxy respondents to answer questions on smoking behaviour for absent individuals | 72292 | Smoking was defined in 2 ways:   1. Currently smoked at least 100 cigarettes and smoking at the time of the interview 2. Smoked daily |
| 8 | Malawi, national, urban and rural | The sampling frame was the list of enumeration areas in Malawi. 144 Enumeration areas were randomly selected from the list of EAs in Malawi. 40 households were then selected from each EA using a systematic sampling method. The sampling interval for selecting households within the EA was determined by dividing the total number of houses in each EA by 40. The Kish Method was then used to select one eligible participant from each household. Households were not replaced if no eligible participants were available in selected households. | 5206 | Tobacco smokers |
| 9 | Nigeria, national urban and rural | The 2006 Population and Housing Census of the Federal Republic of Nigeria was used as the sampling frame for the Nigeria Demographic and Health Survey.  Enumeration areas that provided 888 clusters were stratified by urban and rural areas to allow oversampling of urban areas before selection of households. All women, ages 15–49, who were either permanent residents or overnight visitors in all selected households and all males, ages 15–59, who were either permanent residents or overnight visitors in half of the selected households were eligible to participate. Two NDHS 2008 questionnaires were merged to create the data set for this study | 34070 | Use of smoking tobacco- cigarette, pipe or other |
| 10 | Nigeria, sub-national | Two thirds of the study population was selected from the semi-urban area and the remaining third from the rural community to achieve proportional representation.  A minimum target sample size of 2000 adults aged 25 to 64 stratified by sex and 10 year age group. To compensate for non-response, or attrition, the number was projected to 2200. | 1458 | Tobacco smoking |
| 11 | Rwanda sub-national urban and rural in the capital Kigali urban, and a rural area Huye District | Nyarugenge district is divided into 10 administrative sectors and Huye district has 14 administrative sectors. Each sector has a population register regularly updated. Using population registers systematic sampling was used to obtain probabilistic samples that are representative of the total population. The sample size was estimated at 2138 subjects aged 15 to 80 years (1184 in Kigali and 954 in Huye district). Participants were invited in Health centers where a structured interview was conducted by well-trained health workers. Three health centers were available in Kigali and three others in Huye district. Inclusion criteria were age >15 years, living in Kigali town or Huye District and being competent and willing to sign the informed consent form after being given all the details about the study. | 1920 | Currently smoking at least one cigarette per day |
| 12 | Senegal | A cross-sectional study selected participants using a stratified cluster systematic random sampling method. Conducted in May 2010 in a Senegalese population resident in city of Saint Louis aged 15 years and older. Consent was received. Pregnant women were excluded. | 1424 | Active smoking |
| 13 | Sierra Leone, national | Cross sectional population based survey, all individuals were resident in the study area and willing to participate.  The multi-stage cluster sampling strategy was used in this study. The CSs as demarcated by Statistics Sierra Leone (SSL) were used as the primary sampling unit (PSU). 100 CSs were selected using the probability proportionate to size (PPS) sampling method. Five hundred and fifty EAs were selected from within the selected CSs by the PPS sampling method. In the third stage, ten households were randomly selected from each selected EA. One eligible respondent was selected | 4997 | Current tobacco use (smoke and smokeless) |
| 14 | South Africa/ national | Participants were recruited as part of the first wave of the 2008 South African National Income Dynamics Study (NIDS). The sampling strategy utilised a stratified 2-stage cluster sample to select households. 53 district councils (DC) of South Africa formed the master sample. From within each strata, 400 Primary Sampling Units (PSU) were randomly selected according to the SC PSU allocation. 8 non-overlapping clusters of dwelling units were systematically selected from within in PSU. A household and adult questionnaires were administered | 11,638 | Non-smoker or smoker |
| 15 | Togo/ sub-national | The study was conducted in the town of Lome. 34 households were selected each from 30 clusters to give a total of 1000 households. From each household, one male and one female over 18 years was recruited giving a total of 68 individuals per cluster. Consent was obtained after the study objectives were explained  The sample size of 2000 was calculated to detect a 20% prevalence of hypertension with 80% power at the 0.05 level | 2000 | Consumed at least one cigarette per day |
| 16 | Uganda, sub- national rural | Annual household survey in Southwest Uganda, all household village residents >13 years were eligible to participate | 6678 total  2719 males  3959 females | Current cigarette smoker including both manufactured and local cigarettes |
| 17 | Uganda/sub-national, rural | All residents older than 13 years of age who lived in one half of the rural sub-county. Equal proportions of males and females were sampled. | 6867 | Current daily smoking |
| 18 | Uganda/ sub-national | First 111 villages were randomly selected and stratified by urban or rural residence using the 20002 Uganda Bureau of Statistics. 30 households were selected from each village and Adults Older than 15 years excluding pregnant women who lived in urban and rural communities of Buikwe and Mokono districts in Uganda were recruited after informed consent was obtained. | 4142 | Currently consume tobacco |
| 19 | Zambia/sub-national rural | In the multi-stage sampling procedure, wards were first selected from each constituency. Then standard enumeration areas were selected proportional to the ward size. Households were then systematically sampled from within each standard enumeration area. From within households, all adults older than 25 years were eligible to participate. | 2093 | Current smoking- do you currently smoke any tobacco products such as cigarettes, cigars or pipes |
| 20 | Zambia/sub-national | The mining town of Kitwe in Zambia was first divided into high cost (2) and low cost residential areas (3). One high cost and two lost cost residential areas were randomly selected. The selected areas where then divided into wards where one ward was randomly selected from each constituency. Each selected ward was further divided into Census Supervisory areas where 6 were chosen from the two low cost and four selected from among the high cost residential area. 10 Standard enumeration areas were then selected using probability proportional to size, selecting one each from the selected census supervisory areas. Households were systematically sampled from the chosen standard enumeration areas to ensure a wide coverage among the standard enumeration areas. All adults above 25 years were eligible to participate | 1627 | Current smoking- do you currently smoke any tobacco products such as cigarettes, cigars, or pipes |
| 21 | Zambia, sub-national urban district called Lusaka | Lusaka district was conveniently selected since it was the most urbanised district in Zambia. The hypothesis that that prevalence rates for non-communicable diseases and their risk factors would be highest in urbanized areas. A district was first administratively divided into constituencies, then into wards, Census Standard Areas (CSAs) and finally into Standard Enumeration Areas (SEAs). Lusaka district had 7 constituencies out of which 5 were randomly selected. From each selected constituency, one ward was selected stratified by type of residential area (low, medium and high density areas). CSAs were not considered in our sampling. The number of Standard Enumeration Areas (SEAs) selected in each ward was proportional to its population size. The number of SEAs varied from 15 to 45, and a 1 in 4 systematic random sampling technique was used to select SEAs, except in one ward in which a 1 in 3 systematic method was used. Households were systematically sampled to widely cover the selected SEAs. Finally, all persons of age 25 years or older were requested to participate in the survey. | 1928 | Smoking was defined in 2 ways:   1. Currently smoked cigarettes 2. Do you currently smoke any tobacco products, such as cigarettes, cigars or pipes? |
